# Comprehensive Interactome Analysis for the Sole Adenylyl Cyclase Cyr1 of Candida albicans

**DOI:** 10.1128/spectrum.03934-22

**Published:** 2022-10-31

**Authors:** Guisheng Zeng, Suat Peng Neo, Li Mei Pang, Jiaxin Gao, Shu Chen Chong, Jayantha Gunaratne, Yue Wang

**Affiliations:** a Infectious Diseases Labs, Singapore; b Quantitative Proteomics Group, Institute of Molecular and Cell Biology, Singapore; c Department of Anatomy, Yong Loo Lin School of Medicine, National University of Singapore, Singapore; d Department of Biochemistry, Yong Loo Lin School of Medicine, National University of Singapore, Singapore; University of Guelph

**Keywords:** *Candida albicans*, Cyr1, SILAC, adenylyl cyclase, mass spectrometry

## Abstract

Cyr1, the sole adenylyl cyclase of the fungal pathogen Candida albicans, is a central component of the cAMP/protein kinase A signaling pathway that controls the yeast-to-hypha transition. Cyr1 is a multivalent sensor and integrator of various external and internal signals. To better understand how these signals are relayed to Cyr1 to regulate its activity, we sought to establish the interactome of Cyr1 by using stable isotope labeling by amino acids in cell culture (SILAC)-based quantitative proteomics to identify the proteins that coimmunoprecipitated with Cyr1. The method identified 36 proteins as candidates for authentic Cyr1-interacting partners, together with two known Cyr1-binding proteins, Cap1 and Act1. Fourteen identified proteins belonged to three functional groups, including actin regulation, cell wall components, and mitochondrial activities, that are known to play important roles in cell morphogenesis. To validate the proteomics data, we used biochemical and genetic methods to characterize two cell wall-related proteins, Mp65 and Sln1. First, coimmunoprecipitation confirmed their physical association with Cyr1. Second, deleting either *MP65* or *SLN1* resulted in severe defects in filamentation on serum plates. This study establishes the first Cyr1 interactome and uncovers a potential role for cell wall proteins in directly regulating Cyr1 activity to determine growth forms in C. albicans.

**IMPORTANCE** A critical virulence trait of the human fungal pathogen Candida albicans is its ability to undergo the yeast-to-hypha transition in response to diverse environmental and cellular stimuli. Previous studies suggested that the sole adenylyl cyclase of C. albicans, Cyr1, is a multivalent signal sensor and integrator synthesizing cAMP to activate the downstream hypha-promoting events through the cAMP/protein kinase A pathway. To fully understand how Cyr1 senses and processes multiple stimuli to generate appropriate signal outputs, it was necessary to identify and characterize Cyr1-interacting partners. This study employed SILAC-based quantitative proteomic approaches and identified 36 Cyr1-associated proteins, many having functions associated with hyphal morphogenesis. Coimmunoprecipitation verified two cell surface proteins, Mp65 and Sln1. Furthermore, genetic and phenotypic analyses demonstrated the cAMP-dependent roles of these two proteins in determining hyphal growth. Our study establishes the first Cyr1 interactome and uncovers new Cyr1 regulators that mediate cell surface signals to influence the growth mode of C. albicans.

## INTRODUCTION

Candida albicans is a major opportunistic fungal pathogen of humans. On the one hand, it is a relatively harmless commensal and a member of the normal microbiota in most people’s skin, oral cavity, and gastrointestinal and urogenital tracts. On the other hand, this fungus can cause various superficial infections in otherwise-healthy individuals under favorable conditions. In immunocompromised patients, such as those infected with HIV or undergoing cancer chemotherapy or organ transplantation, C. albicans can disseminate throughout the body to initiate life-threatening systemic diseases with high mortality (1, 2).

C. albicans is a dimorphic fungus and can proliferate in either a unicellular yeast or multicellular filamentous form. It grows as budding yeast-like cells under moderate temperature and low-pH conditions. The yeast cells quickly switch to filamentous growth, forming pseudohyphae or true hyphae, in response to various environmental stimuli, such as temperature elevation to 37°C, neutral pH, and exposure to serum, *N*-acetylglucosamine, or peptidoglycan (3–5). The yeast-hypha transition of C. albicans is closely related to pathogenicity, as mutants locked in either growth form exhibit attenuated virulence in several animal models of infection (6, 7).

The yeast-to-filament growth switch of C. albicans is controlled by multiple regulatory circuits, in particular, the mitogen-activated protein kinase (MAPK) cascade and the cyclic AMP (cAMP)/protein kinase A (PKA) signaling pathway (8, 9). The cAMP/PKA pathway plays a dominant role in serum-induced hyphal growth, as blocking this pathway, but not the MAPK pathway, abolishes true hyphal growth in response to serum induction (6, 10–14). A central component of the cAMP/PKA pathway is Cyr1 (also known as Cdc35), the sole adenylyl cyclase of C. albicans that catalyzes cAMP synthesis (10). cAMP activates PKA, which in turn activates the downstream transcriptional factor Efg1, leading to the expression of hypha-specific genes (8). Cyr1 was first identified by its ability to functionally complement the conditional growth defect of Saccharomyces cerevisiae with mutations in the GTPases Ras1 and Ras2 (10). C. albicans *cyr1*Δ/Δ cells have no detectable intracellular cAMP and fail to undergo hyphal growth under most inducing conditions. However, hyphal growth can be restored in *cyr1*Δ/Δ by adding exogenous cAMP to the medium (10). Cyr1 is a large protein of 1,690 amino acids containing multiple functional domains, and it has been proposed to function as a sensor and integrator of diverse external and internal signals (15, 16). A putative Gα domain near the N terminus of Cyr1 is thought to bind the G-protein α subunit Gpa2 to mediate glucose-induced cAMP signaling, although this interaction has not been demonstrated experimentally (17, 18). Next to the Gα domain is a Ras-association (RA) domain, which interacts with the small GTPase Ras1 to regulate cellular cAMP levels (19). The central region of Cyr1 contains a leucine-rich repeat (LRR) domain capable of recognizing bacterial peptidoglycan (PGN) to stimulate cAMP production and promote hyphal growth (20, 21). The LRR domain may also mediate temperature sensing through interaction with the heat shock protein complex Hsp90/Sgt1 (22, 23). The C-terminal half of Cyr1 harbors the catalytic domain (CYC) that catalyzes cAMP synthesis from ATP. The CYC domain also carries a CO_2_ sensor to mediate CO_2_-induced filamentous growth (21, 24). A putative protein phosphatase 2C domain is located between the LRR and CYC domains, but its function remains unidentified (10). At the extreme C terminus of Cyr1 is a Cap1-binding domain that mediates the formation of a tripartite protein complex containing Cap1, Cyr1, and G-actin (25). This complex has been proposed to sense the dynamic state of the actin cytoskeleton and thus influence the cyclase activity (25). Despite the significant progress in dissecting the role of Cyr1, how this large multivalent protein functions as both signal sensor and integrator remains poorly defined. Therefore, we hypothesized that establishing and functionally characterizing the Cyr1 interactome will provide crucial mechanistic insights.

The stable isotope labeling by amino acids in cell culture (SILAC)-based quantitative proteomics is a powerful tool to establish the interactome of a target protein (26). SILAC is a strategy to label the whole proteome with amino acids metabolically, often lysine and arginine, carrying stable heavy isotopes such as ^13^C or ^15^N, through protein synthesis and turnover during cell growth. When the same proteins from unlabeled (light) and labeled (heavy) samples are combined and analyzed together by high-resolution mass spectrometry (MS), pairs of chemically identical peptides of different stable isotope compositions can be differentiated based on their different masses. The SILAC-based quantitative MS offers a comprehensive yet specific method to identify the interacting partners of a target protein. For example, cells expressing an epitope-tagged bait protein are grown in a medium with heavy amino acids, and cells expressing the epitope alone are grown in a medium with standard amino acids. After protein extraction and immunoprecipitation with an antibody against the epitope, the immunoprecipitates from the two cultures are combined for quantitative MS analysis. Nonspecific binding proteins will have an equal abundance from both light and heavy samples, while specific binding proteins will have a much higher abundance from the heavy sample.

To identify new Cyr1-binding proteins, we constructed a C. albicans strain for efficient labeling with stable isotopes. We then expressed a Myc-tagged Cyr1 in this strain to perform SILAC-based quantitative proteomics. We identified 36 novel Cyr1-interacting partners and validated the interaction with two cell wall proteins, Mp65 and Sln1, by biochemical and genetic methods. Our results establish a comprehensive protein interaction network of Cyr1 and suggest a role of specific cell wall proteins in directly activating the Cyr1-mediated cAMP signaling pathway to influence the growth state of C. albicans.

## RESULTS

### Construction of a C. albicans strain for SILAC-based proteomics.

**FIG 1:**
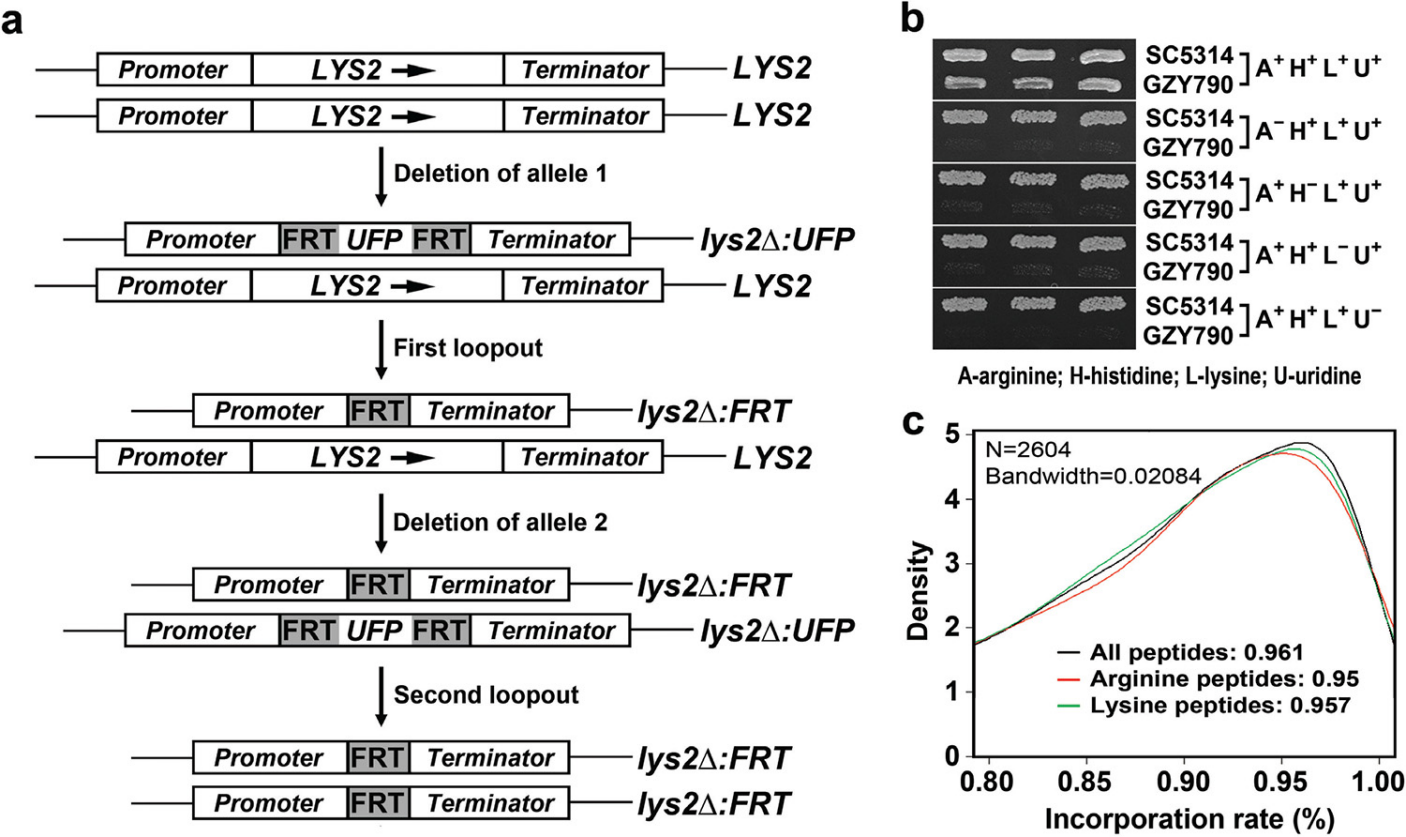
Construction of the strain GZY790 optimized for SILAC experiments. (a) Schematic diagram showing the steps to delete the *LYS2* gene in BWP17 to generate GZY790. UFP, *URA3* flipper; FRT, flippase recognition target. (b) Confirmation of the auxotrophy of GZY790. Cells of GZY790 and SC5314 (WT strain) were grown on a GMM plate supplemented with arginine, histidine, lysine, and uridine and replicated on GMM plates lacking one of the four nutrients as indicated, followed by incubation at 30°C for 40 h. (c) Incorporation rate assay of GZY790. The strain was grown in GMM supplemented with histidine, uridine, and heavy isotope-labeled arginine-d10 and lysine-d8 at 30°C overnight. Cells were then harvested, and protein extracts were prepared to assess the incorporation rate.

To achieve optimal separation between light and heavy peptides during quantitative proteomic analysis, we decided to label the proteome of C. albicans with two heavy amino acids, arginine-d10 (deuterated arginine) and lysine-d8 (deuterated lysine), simultaneously. To generate a strain auxotrophic for both arginine and lysine to allow metabolic labeling, we deleted the two alleles of the *LYS2* gene sequentially (Fig. 1a) in the host strain BWP17 (*ura*Δ *his*Δ *arg*Δ) using the recyclable selectable marker *URA3* flipper (UFP) (27), yielding the strain GZY790 (*ura*Δ *his*Δ *lys*Δ *arg*Δ). We verified the auxotrophy of GZY790 by testing the growth of the strain on media lacking any one of the four amino acids, arginine, histidine, lysine, and uridine (Fig. 1b).

Many factors, such as protein synthesis and degradation, cell division, and growth conditions, may affect the incorporation rate of heavy amino acids into the proteome of a strain. To assess the efficiency of metabolic labeling, GZY790 was cultured in heavy glucose minimal medium (GMM), in which arginine and lysine were provided in the form of arginine-d10 and lysine-d8, at 30°C for 40 h. Protein extracts were then prepared to determine the percentage of incorporation of the heavy amino acids. We found that 95% of peptides were successfully labeled with arginine-d10, while 95.7% were labeled with lysine-d8 (Fig. 1c). When peptides labeled with either arginine-d10 or lysine-d8 or both were counted, the percentage increased to 96.1% (Fig. 1c). This high incorporation rate met the requirement of SILAC-based quantitative proteomic studies. However, when 10 to 20% serum was added to the medium to induce hyphal growth, the incorporation rate of heavy amino acids was reduced to lower than 90%, most likely owing to the presence of natural amino acids in serum, a condition not suitable for the SILAC-based analysis. Thus, our studies described below were carried out only under yeast growth conditions using GMM without serum.

### Purification of Cyr1-associated proteins for SILAC MS.

**FIG 2:**
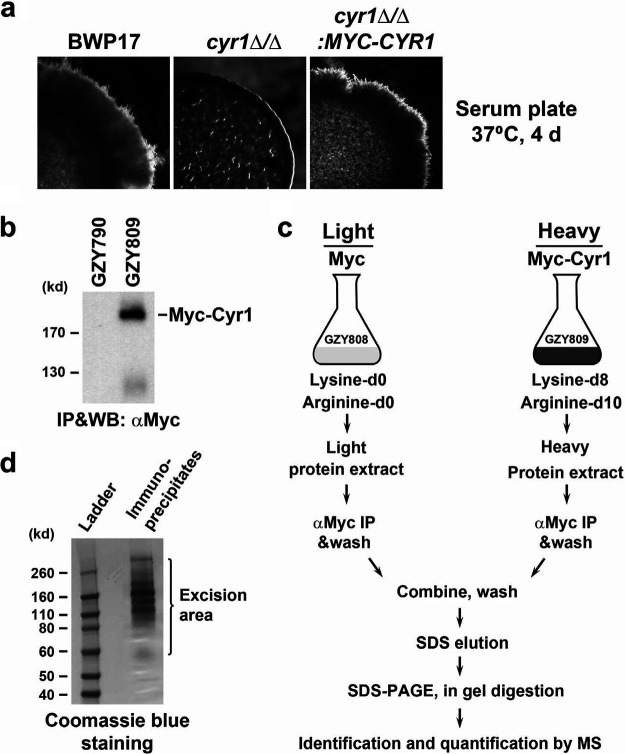
Pulldown of Myc-Cyr1-associated proteins for quantitative analysis. (a) Functional test of Myc-Cyr1. Cultures of BWP17, GZY942 (*cyr1*Δ/Δ), and GZY1519 (*cyr1*Δ/Δ*:MYC-CYR1*) were spotted onto a serum plate and incubated at 37°C for 4 days to allow the formation of filaments along the spot edge. (b) Expression of Myc-Cyr1 in GZY790 for SILAC experiments. *CYR1* in GZY790 was tagged with an N-terminal *MYC* epitope, and expression of Myc-Cyr1 in the resulting strain (GZY809) was confirmed by αMyc IP and WB. (c) Experimental workflow used for the identification and quantification of Myc-Cyr1-associated proteins by SILAC. GZY809 and the control strain GZY808 were grown in heavy and light media, respectively. Cells were harvested and protein extracts were prepared for IP with a Myc antibody conjugated on agarose beads. The beads were combined in a 1:1 ratio during washes. Bead-bound proteins were eluted with SDS and subsequently separated by SDS-PAGE followed by digestion with trypsin for quantitative analysis. (d) Visualization of Myc-Cyr1-associated proteins. The combined immunoprecipitates from GZY808 and GZY809 were separated by SDS-PAGE and stained with Coomassie blue. All visible bands were excised for in-gel trypsin digestion.

To identify Cyr1-associated proteins, we tagged Cyr1 with an N-terminal Myc epitope under the control of the *MET3* promoter in GZY790 to generate the strain GZY809. The Myc tag did not affect the function of Cyr1, since expressing Myc-Cyr1 as the sole adenylyl cyclase restored filamentation to the *cyr1*Δ/Δ mutant (Fig. 2a). The expression of Myc-Cyr1 in GZY809 was confirmed by immunoprecipitation (IP) and Western blotting (WB) with anti-Myc (αMyc) antibodies (Fig. 2b).

Figure 2c schematically illustrates the procedure for quantitatively characterizing Cyr1-interacting partners (see Materials and Methods for details). To metabolically label the whole proteome of GZY809, cells were grown in the heavy medium for 40 h to allow maximum incorporation of heavy amino acids. For the control, a strain expressing only the Myc epitope (GZY808) was cultured in the light medium under otherwise the same conditions. Equal amounts of heavy and light protein extracts were prepared and incubated with agarose beads conjugated with the Myc antibody separately. The beads from the two samples were then combined for washes. Finally, the bead-bound proteins were eluted with SDS, separated by SDS-PAGE, and visualized with Coomassie blue staining (Fig. 2d). All visible protein bands were excised, subjected to in-gel trypsin digestion, and analyzed using a nanoLC Orbitrap MS system.

### Identification of Cyr1-binding partners by quantitative analysis.

**FIG 3:**
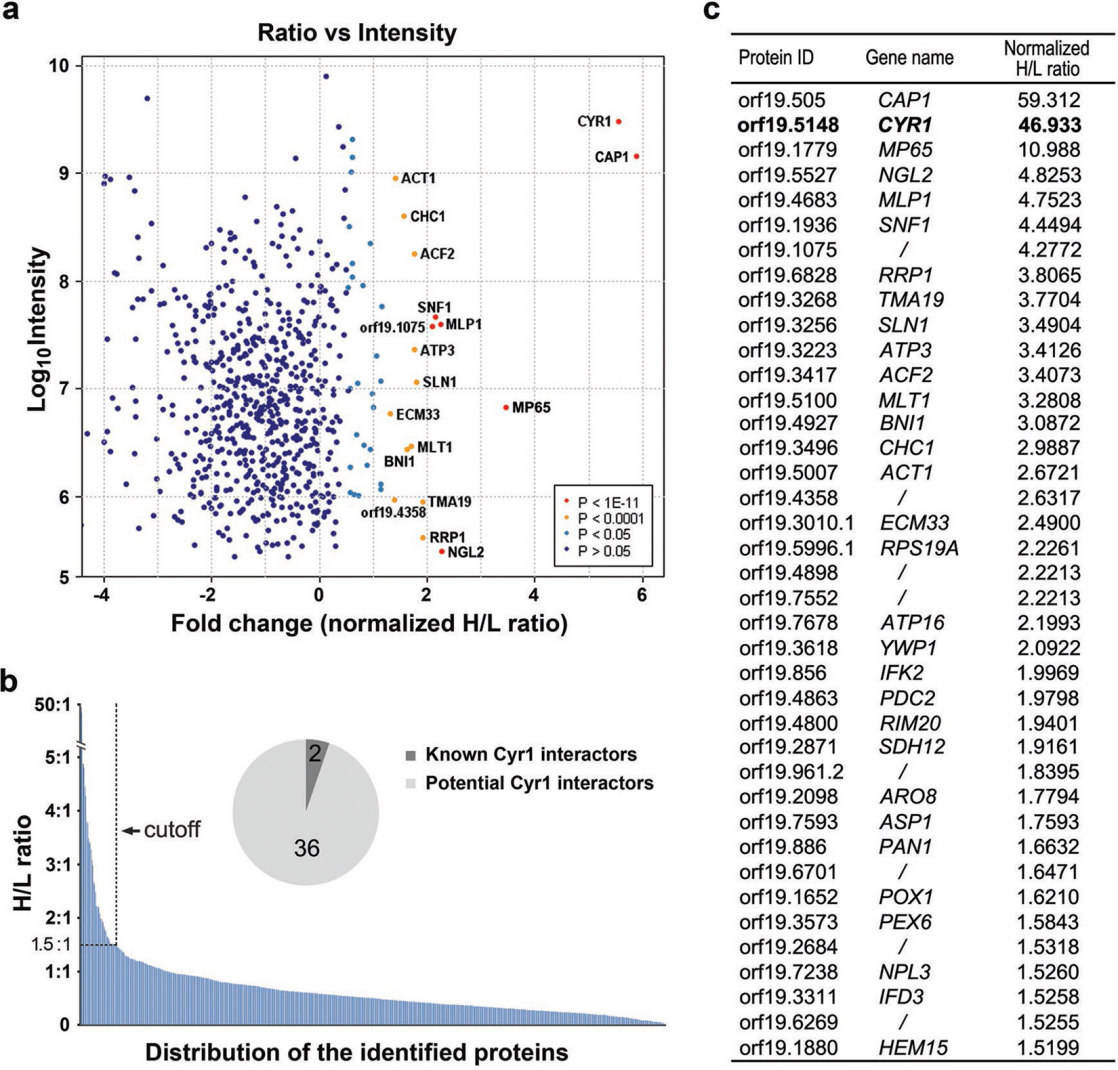
Quantitative analysis of Myc-Cyr1-associated proteins. (a) Ratio versus intensity plot of identified Myc-Cyr1-associated proteins. The trypsin-digested immunoprecipitates from GZY808 and GZY809 were processed for protein identification and quantitation. Each protein’s heavy:light (H/L) ratio was plotted against its total intensity (in log_10_). Red and yellow dots (labeled with protein ID) indicate the potential interactors of Cyr1. (b) Distribution of the identified proteins according to their normalized H/L ratios. Proteins associated with an H/L ratio of more than 1.5:1 were considered potential Cyr1 interactors. The pie chart represents the proportion of previously known and newly identified Cyr1-interacting proteins. (c) List of the protein identities of potential Cyr1 interactors with the corresponding gene names and normalized H/L ratios. The bait protein Cyr1 is highlighted in bold.

A total of 670 proteins were identified from the immunoprecipitates of Myc-Cyr1 (see Table S1 in the supplemental material). SILAC MS made it possible to compare the amounts of proteins immunoprecipitated from cells expressing Myc (GZY808, light) or Myc-Cyr1 (GZY809, heavy). The integrated intensity sums of the peptide peaks determined by MaxQuant reflected the peptide abundance, and the fold change represented the ratio of peptides quantified between the experimental sample labeled with heavy amino acids and the control sample labeled with light amino acids. A plot of ratio versus log_10_ intensity was then drawn to display all the identified proteins as dots on a map. The identities of the proteins with the highest possibility of being Cyr1 interactors were labeled with yellow and red dots (Fig. 3a). Many proteins identified by SILAC MS exhibited a normalized heavy:light (H/L) ratio below 1:1 (Fig. 3b), likely representing the background or nonspecific protein interactions. The greater a peptide H/L ratio’s deviation from 1:1, the higher the likelihood it is a true Cyr1-binding partner. By using an H/L ratio of 1.5:1 as a cutoff, we identified 38 high-priority Cyr1-interacting proteins (Fig. 3c), including two previously characterized Cyr1-associated proteins, Cap1 (normalized H/L ratio of 59) and Act1 (normalized H/L ratio of 2.7) (25).

### Physical and genetic interactions between Mp65 and Cyr1.

Identifying Cap1 and Act1 among the top candidates for Cyr1 interactors provided strong validation for the SILAC-based method. Among the remaining 36 highly confident candidates, Mp65 had the highest normalized H/L ratio, ~11 (Fig. 3c). This protein is a putative β-glucanase mannoprotein and has been previously reported to have a role in several virulence-related traits, such as cell wall integrity, adhesion, biofilm formation, hyphal morphogenesis, and pathogenicity (28, 29). Therefore, we selected Mp65 for further investigation.

**FIG 4:**
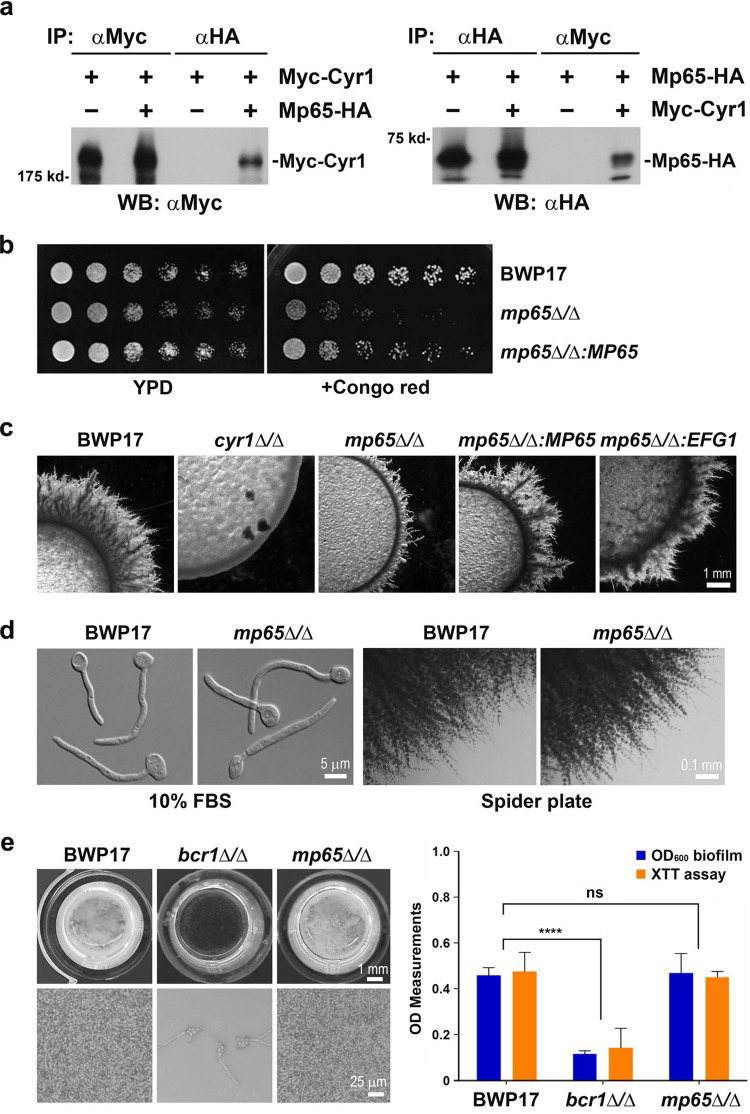
Physical and genetic interactions between Mp65 and Cyr1. (a) Validation of the physical interaction between Cyr1 and Mp65 by co-IP. Protein extracts prepared from GZY835 (Myc-Cyr1), GZY865 (Mp65-HA), and GZY866 (Myc-Cyr1 Mp65-HA) were subjected to αHA and αMyc IP, followed by SDS-PAGE and WB with the αMyc (left) and αHA (right) antibodies. (b) Congo red sensitivity test of the *mp65*Δ/Δ mutant. YPD cultures of BWP17, *mp65*Δ/Δ (GZY871), and *mp65*Δ/Δ*:P_MET3_-GFP-MP65* (GZY900) were serially diluted (1:10) in water and spotted onto YPD plates containing 0 or 100 μg/mL Congo red. The plates were incubated at 30°C for 24 h. (c) Defective filamentation of the *mp65*Δ/Δ mutant on serum plate. YPD cultures of BWP17, *cyr1*Δ/Δ, *mp65*Δ/Δ, *mp65*Δ/Δ*:P_MET3_-GFP-MP65*, and *mp65*Δ/Δ*:TetOff-Myc-EFG1* (GZY1521) were spotted onto serum plates and incubated at 37°C for 4 days. (d) Hyphal growth of *mp65*Δ/Δ in liquid culture and filamentous growth of *mp65*Δ/Δ on Spider plate. Cultures of BWP17 and *mp65*Δ/Δ were induced for hyphal growth in YPD containing 10% FBS at 37°C for 2 h. For filamentous growth on Spider plates, YPD cultures of BWP17 and *mp65*Δ/Δ were streaked onto Spider plates and incubated at 30°C for 6 days. (e) Biofilm development assays of the *mp65*Δ/Δ mutant. BWP17, *bcr1*Δ/Δ (GZY1094), and *mp65*Δ/Δ were grown on the bottom of a 96-well polystyrene plate to induce biofilm formation. Biofilms of each strain were subjected to visual inspection and microscopic examination and further quantified by density measurement at OD_600_ and an XTT assay at OD_490_. BWP17 formed normal biofilms and *bcr1*Δ/Δ formed defective biofilms. ****, *P < *0.0001. *mp65*Δ/Δ displayed no significant (ns) differences in OD_600_ (*P = *0.7441) or the XTT assay (*P = *0.4147) compared to BWP17.

To confirm that Mp65 is an authentic Cyr1-binding partner, we tagged Mp65 with a C-terminal hemagglutinin (HA) epitope and coexpressed it with Myc-Cyr1 for co-IP experiments. When an αHA antibody was used to pull down Mp65-HA from cell lysates, Myc-Cyr1 was detected in the IP product by αMyc WB (Fig. 4a, left). Reciprocally, when Myc-Cyr1 was precipitated with an αMyc antibody, Mp65-HA was detected by αHA WB (Fig. 4a, right). In the control experiments, the αHA antibody failed to pull down Myc-Cyr1 from cells expressing Myc-Cyr1 alone, and the αMyc antibody failed to pull down Mp65-HA from cells where Cyr1 was untagged (Fig. 4a). These experiments demonstrated that Cyr1 and Mp65 physically interact with each other *in vivo*.

To explore the functional relationship between Mp65 and Cyr1 in regulating hyphal growth, we generated an *mp65*Δ/Δ mutant using the common laboratory strain BWP17. The two wild-type (WT) copies of *MP65* were replaced by the auxotrophic markers *UFP* and *HIS1*, and the deletion of *MP65* was verified by colony PCR according to standard protocols (30). Consistent with a previous study (28), our *mp65*Δ/Δ mutant was sensitive to the cell wall-perturbing agent Congo red (CR) (Fig. 4b). When the culture of *mp65*Δ/Δ was serially diluted and spotted onto yeast extract-peptone-dextrose (YPD) plates, the cells grew well in the absence of CR but failed to grow on plates containing 100 μg/mL CR. In comparison, the growth of BWP17 cells was unaffected by CR. When a WT copy of *MP65* was reintroduced into the *mp65*Δ/Δ mutant, the CR sensitivity was completely rescued, indicating that the cell wall defect of *mp65*Δ/Δ was caused by the loss of *MP65*. These results confirmed that *MP65* is required for cell wall integrity.

Next, we tested the *mp65*Δ/Δ mutant together with the *cyr1*Δ/Δ mutant for filamentous growth under various growth conditions. Interestingly, we found that the filamentous growth of *mp65*Δ/Δ was severely compromised, although not totally abolished as with *cyr1*Δ/Δ, on the serum plate (Fig. 4c). The filamentation defect was rescued by either reintegrating into the mutant a WT copy of *MP65* or by overexpressing the hyphal transcription factor Efg1 (Fig. 4c). However, the *mp65*Δ/Δ mutant exhibited normal hyphal growth, like BWP17, in the liquid YPD+serum medium (Fig. 4d), which contradicted a previous report (29). When cultured in YPD containing 10% serum at 37°C for 2 h, *mp65*Δ/Δ cells produced thin, long hyphae morphologically indistinguishable from those of BWP17. The *mp65*Δ/Δ mutant also underwent normal filamentous growth on a Spider plate (Fig. 4d). After 6 days of incubation at 30°C on a Spider plate, filaments with short branches of comparable lengths were seen along the edges of both *mp65*Δ/Δ and BWP17 colonies. In addition, the *mp65*Δ/Δ mutant exhibited normal biofilm formation, like BWP17 (Fig. 4e). After incubation in the GMM at 37°C for 48 h in a flat-bottomed 96-well polystyrene plate, both strains formed healthy biofilms with comparable architecture and quantity, which was in sharp contrast with that of the biofilm-defective strain *bcr1*Δ/Δ (31) (Fig. 4e).

Nevertheless, the physical interaction between Mp65 and Cyr1, the common filamentation defect shared by *mp65*Δ/Δ and *cyr1*Δ/Δ mutants on serum plates, and the suppression of *mp65*Δ/Δ filamentation defect by Efg1 overexpression all suggest that Mp65 directly interacts with Cyr1 to activate the cAMP/PKA pathway to regulate filamentous growth under certain conditions.

### Physical and genetic interactions between Sln1 and Cyr1.

**FIG 5:**
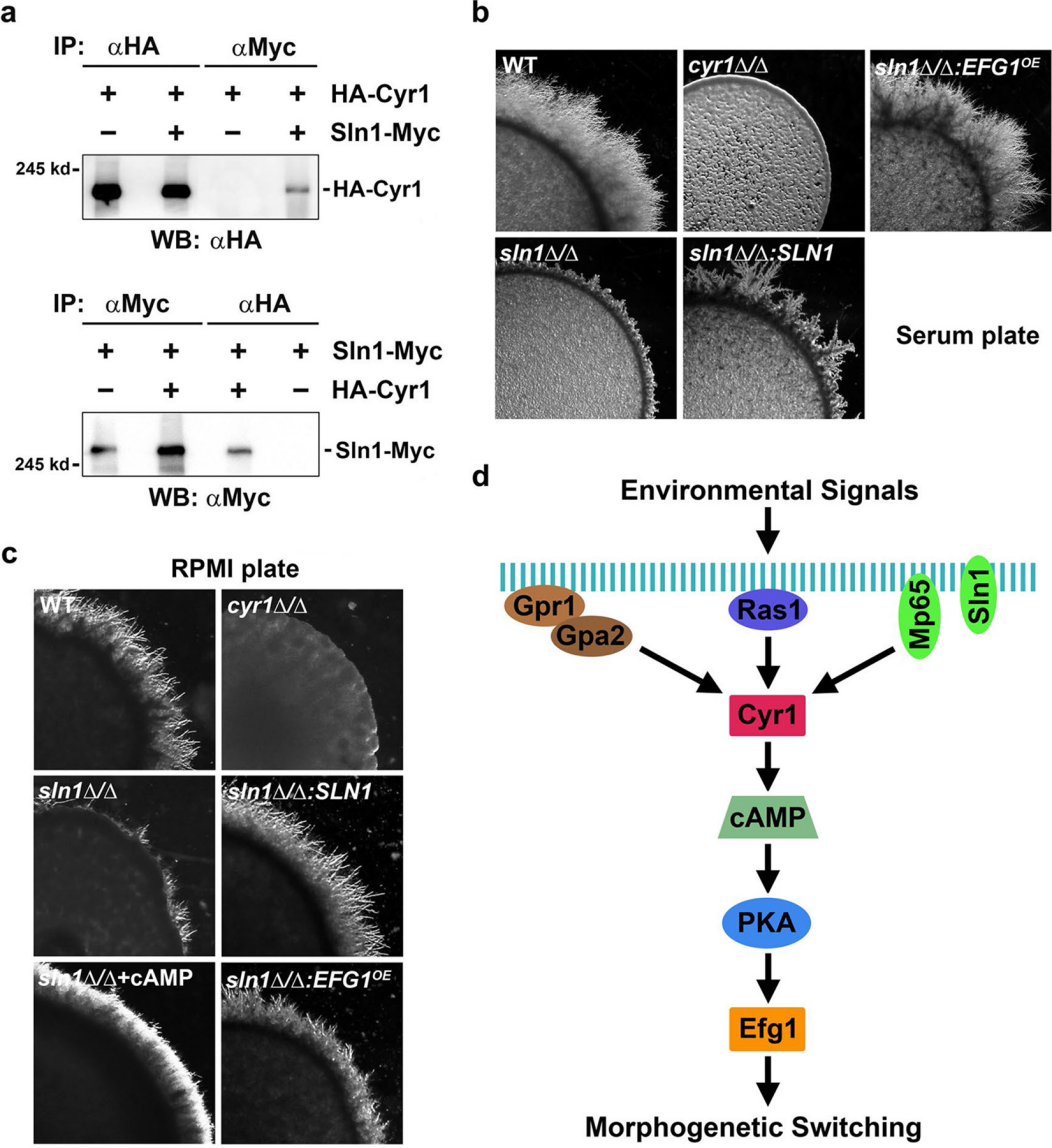
Physical and genetic interactions between Sln1 and Cyr1. (a) Validation of the physical interaction between Sln1 and Mp65 by co-IP. Protein extracts prepared from GZY847 (Sln1-Myc), GZY855 (HA-Cyr1), and GZY851 (HA-Cyr1 Sln1-Myc) were subjected to αHA and αMyc IP, followed by SDS-PAGE and WB with the αMyc and αHA antibodies. (b) Defective filamentation of the *sln1*Δ/Δ mutant on serum plate. YPD cultures of BWP17, *cyr1*Δ/Δ, *sln1*Δ/Δ (GZY1512), *sln1*Δ/Δ*:SLN1-Myc* (GZY1518), and *sln1*Δ/Δ*:TetOff-Myc-EFG1* (GZY1520) were spotted onto serum plates and incubated at 37°C for 5 days. (c) Defective filamentation of the *sln1*Δ/Δ mutant on RPMI plate. YPD cultures of BWP17, *cyr1*Δ/Δ, *sln1*Δ/Δ, *sln1*Δ/Δ*:SLN1-Myc*, and *sln1*Δ/Δ*:TetOff-Myc-EFG1* were spotted onto RPMI plates and incubated at 37°C for 3 days. In addition, the *sln1*Δ/Δ mutant was also tested for filamentous growth on a RPMI plate containing 20 mM cAMP. (d) Schematic diagram depicting how Sln1 (and Mp65) participates in the regulation of filamentous growth via the physical interaction with Cyr1.

In addition to Mp65, we also chose Sln1, a cell surface histidine kinase involved in a two-component signaling pathway and cell wall biosynthesis (32), to validate its physical interaction and functional relationship with Cyr1. As demonstrated by co-IP experiments, the αMyc antibody pulled down HA-Cyr1 only in the presence of Sln1-Myc (Fig. 5a), and reciprocally, the αHA antibody pulled down Sln1-Myc only in the presence of HA-Cyr1 (Fig. 5a). These results suggest that Sln1 is another authentic Cyr1 interactor.

Next, we constructed an *sln1*Δ/Δ mutant to investigate the functional relationship between Sln1 and Cyr1. Like *mp65*Δ/Δ, the *sln1*Δ/Δ mutant exhibited a severe defect in filamentation on serum plates. This defect was largely rescued by reintroducing a WT copy of *SLN1* and by overexpressing *EFG1* (Fig. 5b). In addition, the *sln1*Δ/Δ mutant also showed a severe filamentous growth defect on RPMI plates, which was largely corrected by a WT copy of *SLN1* and suppressed by either adding cAMP to the medium or overexpressing *EFG1* (Fig. 5c).

Together, these data suggest that Sln1, like Mp65, also participates in the cAMP/PKA pathway to regulate filamentation in certain conditions via physical association with Cyr1 (Fig. 5d).

### Classification of the identified Cyr1-binding partners.

**FIG 6:**
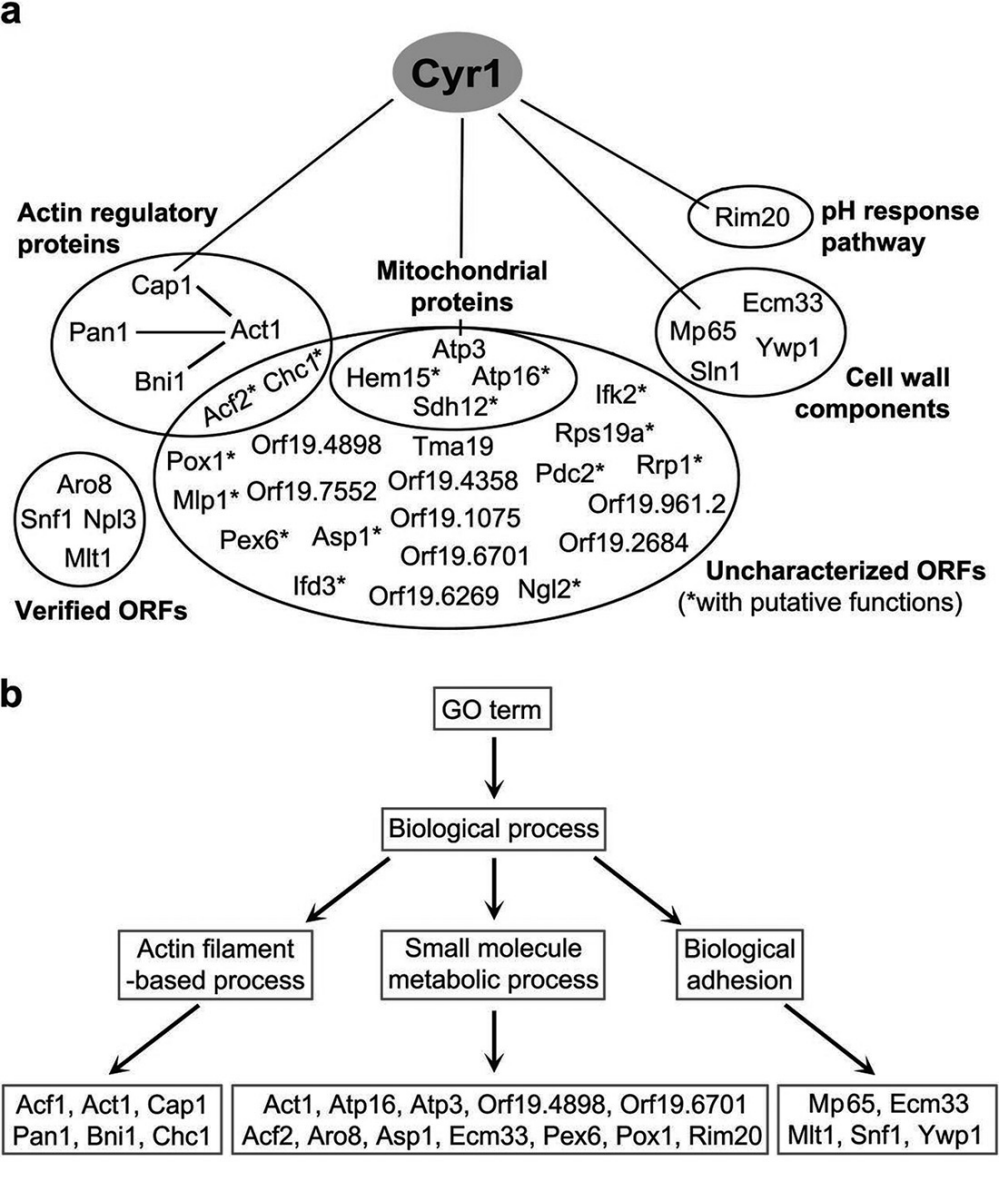
Classification of potential Cyr1 interactors identified by the SILAC-based proteomic approach. (a) The 36 potential Cyr1 interactors with two known binding partners (Cap1 and Act1) were manually curated into different groups. Some proteins were classified into more than one group. (b) Query of the Cyr1 potential interactors with Gene Ontology (GO) Term Finder (*Candida* Genome Database) under “Biological process” ontology classified some of the candidates into three categories: actin filament-based process, small molecule metabolic process, and biological adhesion.

The experimental confirmation of Mp65 and Sln1 as authentic Cyr1 interactors and their functional relationship with Cyr1 in regulating filamentous growth support the rest of the SILAC-identified Cyr1-associated proteins being members of the Cyr1 interactome. To better interpret the proteomics data, we classified the 38 Cyr1-interacting proteins (including the two known interacting partners, Cap1 and Act1) into several groups, based on their molecular functions and biochemical properties, with an emphasis on established protein-protein interactions (Fig. 6).

When we analyzed the molecular functions of the potential Cyr1 interactors using the Gene Ontology Term Finder from the *Candida* Genome Database, no significant ontology term was found for the input genes. Therefore, we conducted manual curation (Fig. 6a). Among the 38 identified proteins, more than half of the candidates were uncharacterized and therefore classified into a group of “uncharacterized ORFs.” In this group, some proteins had putative functions inferred from their characterized orthologs in other species (marked with an asterisk). Four proteins within this group (Atp3, Atp16, Hem15, and Sdh12) had mitochondrion-related functions and formed a subgroup of “mitochondrial proteins.” Four actin-related proteins, Bni1, Pan1, Chc1, and Acf2, together with Cap1 and Act1, formed an “actin regulatory proteins” group. These proteins either bind to actin or regulate cortical actin patch dynamics (25, 33–37). We classified Mp65 with Sln1, Ywp1, and Ecm33 into a “cell wall components” group, as these proteins either localize to the cell wall and/or regulate cell wall biosynthesis (28, 32, 38–40). Rim20 is the only potential Cyr1-interacting partner that functions in the pH response pathway (41). Four verified open readings frames (ORFs; Aro8, Snf1, Npl3, and Mlt1) did not belong to any of the above groups and thus were classified as “verified ORFs.”

To identify possible biological processes involving the potential Cyr1-binding partners, we also input the 38 candidates to the Gene Ontology Term Finder and performed a search using the default setting. In addition to the “small molecule metabolic process,” two prominent biological processes were identified: one was “actin filament-based process,” which consisted of Act1, Bni1, Cap1, Pan1, Chc1, and Acf2, and the other was “biological adhesion,” that contained Mp65, Ecm33, Ywp1, Mlt1, and Snf1 (Fig. 6b). These findings are largely consistent with the manual classification above.

## DISCUSSION

As the sole adenylyl cyclase of the human fungal pathogen C. albicans, Cyr1 catalyzes cAMP synthesis in response to many external and internal stimuli to trigger the cAMP-dependent signaling pathway for proper physiological responses (42). Although several factors that regulate the catalytic activity of Cyr1 have been characterized (20, 24, 43), how Cyr1 senses and integrates the wide range of external and internal signals with distinct natures remains largely unknown. In this work, we applied SILAC-based proteomic approaches to establish the interactome of Cyr1. We identified 36 novel candidates as potential interactors of Cyr1 and validated two by biochemical and genetic methods, thus providing a valuable resource for discovering novel molecular mechanisms that regulate Cyr1 activities in response to various stimuli.

### The SILAC-optimized C. albicans strain GZY790.

Since its development in 2002, SILAC-based quantitative proteomic analysis has become an excellent, popular technique to reveal changes in protein abundance and identify protein interaction partners in many organisms. The metabolic labeling of SILAC has proven to have unique and specific advantages compared to other labeling methods. However, SILAC has limitations in its application, because human tissue and organ samples and some types of unicellular organisms may encounter difficulties in incorporating heavy amino acids. Until recently, the utilization of SILAC to C. albicans had been proven applicable and used to identify interactors of the protein phosphatase Cdc14 (44, 45).

For a successful SILAC experiment, the choice of heavy isotope-labeled amino acids is crucial. The heavy amino acids are required to provide at least 4-Da separation of heavy and light peptides to minimize the overlapping of light and heavy peptide clusters and inaccuracy in quantitation. The commonly used laboratory strain of C. albicans, BWP17, is only suitable for metabolic labeling with heavy arginine, since it is auxotrophic for uridine, histidine, and arginine (46). To ensure the resolution of heavy and light peptide pairs, we further introduced lysine auxotrophy into BWP17 by deleting the *LYS2* gene to generate the strain GZY790. Being auxotrophic for both arginine and lysine, GZY790 allowed the incorporation of heavy arginine and lysine into the C. albicans proteome simultaneously at a rate higher than 95%, rendering it a useful tool strain for SILAC-based quantitative proteomics. The successful identification of new Cyr1-interacting partners with two known ones using GZY790 demonstrates the efficacy of this strain.

### The identification of novel Cyr1-interacting proteins.

Combining SILAC-based metabolic labeling and quantitative MS analysis enabled us to identify the proteins that coimmunoprecipitated with Myc-Cyr1 and exclude the nonspecific binding proteins. The nonspecific binding proteins were most likely pulled down during IP due to their association with the Myc epitope, the Myc antibody, and the antibody-conjugated beads rather than with Cyr1 itself, thus showing a similar abundance in light and heavy samples. It was expected that the SILAC MS method would identify genuine Cyr1-interacting proteins. Indeed, we identified Cap1 and Act1 with high confidence, two proteins previously shown to form a stable complex with Cyr1 (25). We also discovered 36 more as potentially novel Cyr1-interacting proteins, revealing a comprehensive interactome for Cyr1. One of the novel proteins, Snf1, has been confirmed to physically interact with Cyr1 in S. cerevisiae (47). However, we failed to detect some other proteins previously shown or thought to interact with Cyr1 by other methods, such as the small GTPase Ras1 (19). Two possible explanations are that (i) the interaction between Ras1 and Cyr1 requires activation by external hyphal induction signals, such as serum, while our experiments were done in yeast cells. (ii) Second, the association of these two proteins is transient and, therefore, difficult to detect. This may also explain why this experiment did not pick up Gpa2 and Hsp90/Sgt1. The association of Gpa2 with Cyr1 may require external inducers, such as glucose or amino acids (17, 18), while the association of Sgt1 with Cyr1 may happen only at elevated temperatures (22). Therefore, it is necessary to perform the same experiment under different growth conditions, such as filamentous growth induced by various stimuli, to construct a more comprehensive interactome for Cyr1.

### Actin regulatory proteins as Cyr1 interactors.

Among the identified novel Cyr1 interactors, several (Pan1, Bni1, Chc1, and Acf2) are functionally related to actin dynamics. Together with Cap1 and Act1, these proteins form the group of actin regulatory proteins. Pan1 functions as an activator of the actin-nucleating Arp2/3 complex in S. cerevisiae (33) and has been shown to form a complex with the endocytic protein Sla1 to regulate cortical actin patch dynamics during the polarized growth of C. albicans (36). Bni1 is the yeast formin that mediates actin filament assembly independent of the Arp2/3 complex (34). In C. albicans, Bni1 plays an important role in cell polarity control during both yeast and hyphal growth (48). Although the functions of Chc1 and Acf2 have not been characterized in C. albicans, the ortholog of Chc1 (clathrin heavy chain) in S. cerevisiae has been demonstrated to associate with the actin-based endocytic machinery (35). The ortholog of Acf2 (assembly complementing factor) in S. cerevisiae is required for *in vitro* cortical actin assembly (37). We noticed that two more actin-related proteins, Sla2 and Arc18, were also identified in the same experiment, but with a normalized H/L ratio (1.4153 and 1.177, respectively) that was slightly lower than the cutoff value we used (see Table S1). In C. albicans, Sla2 localizes to cortical actin patches and is required for the proper organization of the actin cytoskeleton and formation of true hyphae in response to serum and other stimuli (49, 50). The ortholog of Arc18 in S. cerevisiae is a subunit of the Arp2/3 complex that is required for the motility and integrity of cortical actin patches (51).

Because Cyr1 forms a stable tripartite complex with Act1 via Cap1 (25), it is unsurprising that some actin-associated proteins were coimmunoprecipitated with Cyr1. However, we cannot rule out the possibility that some actin regulatory proteins may interact with Cyr1 directly, independent of Cap1 and Act1. What are the physiological roles of the interaction between Cyr1 and actin regulatory proteins? We previously proposed that the Cyr1/Cap1/actin complex may provide a mechanism that monitors the intracellular status of the actin cytoskeleton and adjust cAMP synthesis to best regulate actin-mediated cellular processes, such as polarized growth (25, 52). Here, based on the discovery of more Cyr1-associated actin regulators, we expanded our hypothesis that Cyr1 might directly interact with these proteins in organizing the actin cytoskeleton for polarized growth in response to external and internal stimuli. Because cortical actin patches are the active endocytic sites, it is also possible that these actin regulatory proteins may help to internalize some external inducers through the endocytic pathway and subsequently deliver the signals to Cyr1.

### Mp65, Sln1, and cell wall components as Cyr1 interactors.

In addition to actin regulatory proteins, our experiments also identified four proteins (Mp65, Sln1, Ecm33, and Ywp1) with cell wall-related functions. Mp65 is a putative β-glucanase adhesin previously shown to be required for cell wall integrity and several infection-related cellular processes (28, 29). Sln1 is a histidine kinase involved in a two-component signaling pathway regulating cell wall biosynthesis (32). Ywp1 is a yeast cell wall protein that appears to be linked covalently to the glucan of the cell wall matrix (38). Ecm33 is a glycosylphosphatidylinositol-anchored protein required for cell wall integrity, morphogenesis, and virulence of C. albicans (39). Together, these proteins define a novel group of Cyr1 interactors: cell wall components.

The cell surface localization of these cell wall proteins suggests a possible role in sensing and transmitting external signals to Cyr1. We confirmed the association of Mp65 and Sln1 with Cyr1 by co-IP experiments, thus revealing a novel link between cell surface proteins and the intracellular Cyr1. Sln1 had been predicted to be a cell surface protein with an extracellular sensor domain (53). By transmembrane topology analysis (http://www.ch.embnet.org/software/TMPRED_form.html), we also identified several highly possible transmembrane domains in Mp65 with regions exposed on both the outside and inside of the plasma membrane, providing potential binding sites for signaling molecules and association with Cyr1 (Fig. S1). It is therefore tantalizing to propose that Mp65 and Sln1 transduce external signals across the plasma membrane to regulate Cyr1 activity. In support of this hypothesis, two recent studies have demonstrated that Cyr1 is associated with the peripheral cell membrane (54, 55).

In addition, we found that both *mp65*Δ/Δ and *sln1*Δ/Δ mutants were defective in filamentous growth under certain conditions, although the defects were not as severe as that of the *cyr1*Δ/Δ mutant. More importantly, such defects could be suppressed by the overexpression of Efg1 (and the addition of exogenous cAMP in the case of *sln1*Δ/Δ), the transcription factor downstream of Cyr1 in the cAMP/PKA pathway. Therefore, we propose that the binding of Mp65 and Sln1 to Cyr1 may stabilize and even enhance the activity of Cyr1, leading to the activation of the cAMP/PKA pathway and stimulation of filamentation under certain circumstances.

Since Mp65 has been demonstrated to be a major antigen target of the cell-mediated immune response to C. albicans (56), it is reasonable to speculate that Mp65 (and possibly other cell wall proteins) may trigger the hyphal growth of C. albicans via the cAMP pathway to protect the pathogen against attacks by the human immune system. If this is true, Mp65 and other cell wall proteins identified in this study may provide a novel group of targets for both pharmacological and immunological antifungal therapies.

### Rim20 as a Cyr1 interactor.

pH is an important environmental variable that affects C. albicans morphogenesis, with alkaline conditions favoring hyphal growth and acidic conditions favoring yeast growth (57). C. albicans pH sensing and its morphological response to pH is primarily mediated by the Rim101 signal transduction pathway (41). Recently, the cAMP/PKA signaling pathway was shown to be involved in the pH-dependent morphological responses of C. albicans (16). It was reported that acidic pH downregulated cAMP signaling and inhibited hyphal growth in minimal medium containing GlcNAc. More importantly, such an effect is thought to be mediated, at least partially, through Cyr1 in a Ras1-independent fashion. In other words, low pH inhibits cAMP synthesis by Cyr1 independently of Ras1 to negatively regulate hyphal growth (16). However, how the catalytic activity of Cyr1 is downregulated by low pH to reduce cAMP synthesis remains unknown. The identification of Rim20 as a novel Cyr1-interacting protein in this study offers a possible molecular mechanism to solve this mystery. Rim20 participates in the pH response pathway and binds to the transcription factor Rim101 to facilitate the proteolytic activation of Rim101 in alkaline pH (41). Rim20 may similarly bind to Cyr1 in low pH and inactivate its catalytic activity by proteolytic cleavage.

### Mitochondrial proteins as Cyr1 interactors.

It has been known for a long time that the absence of some mitochondrial proteins, such as the NADH dehydrogenase Ndh51 and the pyruvate dehydrogenase complex protein X Pdx1, result in defective filamentous growth under certain conditions (58, 59). In this study, four mitochondrion-related proteins, including Atp3, Atp16, Sdh12, and Hem15, were identified as novel interactors of Cyr1. Atp3 and Atp16 are subunits of the mitochondrial ATP synthase localized to the mitochondrial inner membrane (60). Sdh12 is the homolog to yeast Sdh1, a flavoprotein succinate dehydrogenase that couples the oxidation of succinate to the transfer of electrons to ubiquinone as part of the tricarboxylic acid cycle and the mitochondrial respiratory chain (61). Hem15 is a mitochondrial inner membrane protein that participates in heme biosynthesis (62). In support of our finding, a functional link between Cyr1 and mitochondrial respiration has been demonstrated in S. cerevisiae (63), and Cyr1 has been shown to function together with mitochondria to regulate Ras1 activity in C. albicans (64).

In summary, using SILAC-based quantitative proteomics, we identified 36 novel Cyr1-associated proteins. Six actin regulatory proteins may function as either the upstream regulators of Cyr1 to control its cyclase catalytic activity or downstream effectors to organize the actin cytoskeleton for polarized growth. Four cell wall proteins may help to stabilize and/or enhance the Cyr1-dependent cAMP pathway to initiate the switch from yeast to hyphal growth in response to external signals, such as attacks by the host immune system. The identification of Rim20 may explain how the catalytic activity of Cyr1 is downregulated by low pH. Interaction of four mitochondrial proteins with Cyr1 may coordinate mitochondrial activity with Ras1-GTP binding. Many other proteins identified in this study remain uncharacterized, and the interaction with Cyr1 may provide a starting point for their functional characterization. Future studies are required to validate the physical interactions of these potential binding partners with Cyr1 by other independent methods, such as co-IP, yeast two-hybrid assays, and *in vitro* binding assays, and reveal the functional relationships. It is also important to identify the domains of Cyr1 for each of the interactions. Undoubtedly, establishing a comprehensive interacting network for Cyr1 will greatly advance our understanding of the regulation and function of this import fungal adenylyl cyclase.

## MATERIALS AND METHODS

### Strains, plasmids, and growth conditions.

Yeast strains and plasmids used in this study are described in Tables 1 and 2, respectively. Recombinant DNA manipulations were performed according to standard methods. E. coli XL1-Blue (Stratagene) was used as the host strain for recombinant plasmids and cultured in LB broth (0.5% yeast extract, 1% tryptone, and 0.5% NaCl; pH 7.0) supplemented with 100 μg/mL ampicillin. Transformation of C. albicans was performed according to the protocol of the Fast Yeast transformation kit (G-Biosciences). Gene deletion was verified by colony PCR as described elsewhere (30). Looping out of *URA3* via FLP-mediated excision followed previous protocols (27). Yeast cells were routinely grown at 30°C in YPD (2% yeast extract, 1% peptone, and 2% glucose) or GMM (glucose minimal medium; 6.79 g/liter yeast nitrogen base without amino acids, 2% glucose) supplemented with amino acids and other compounds (80 μg/mL uridine, 40 μg/mL arginine, 40 μg/mL histidine, 50 μg/mL lysine, and 1 mg/mL 5-fluoroorotic acid) when necessary. Solid plates were prepared by adding 2% agar to the liquid media. Serum plates contained 10% fetal bovine serum (FBS) and 2% agar. Spider plates contained 10 g/liter nutrient broth, 10 g/liter mannitol, 2 g/liter K_2_HPO_4_, and 2% agar (pH 7.2). RPMI plates were prepared by adding 2% agar to liquid RPMI 1640 medium (Gibco).

**TABLE 1:** C. albicans strains used in this study

Strain	Relevant genotype
BWP17	*ura3*::*imm434/ura3*::*imm434 his1*::*hisG/his1*::*hisG arg4*::*hisG/arg4*::*hisG*
GZY790	BWP17, *lys2Δ*::*FRT/lys2Δ*::*FRT*
GZY808	GZY790, *P_MET3_-Myc-URA3*
GZY809	GZY790, *CYR1/cyr1*::*P_MET3_-Myc-CYR1-URA3*
GZY835	BWP17, *CYR1/cyr1*::*P_MET3_-Myc-CYR1-URA3*
GZY847	BWP17, *SLN1/sln1*::*SLN1-Myc-URA3*
GZY851	BWP17, *CYR1/cyr1::P_MET3_-HA-CYR1-ARG4 SLN1/sln1*::*SLN1-Myc-URA3*
GZY855	BWP17, *CYR1/cyr1*::*P_MET3_-HA-CYR1-ARG4*
GZY865	BWP17, *MP65/mp65*::*MP65-HA-HIS1*
GZY866	BWP17, *CYR1/cyr1*::*P_MET3_-Myc-CYR1-URA3 MP65/mp65*::*MP65-HA-HIS1*
GZY871	BWP17, *mp65Δ*::*UFP/mp65Δ*::*HIS1*
GZY888	BWP17, *mp65Δ*::*FRT/mp65Δ*::*HIS1*
GZY900	BWP17, *mp65Δ*::*FRT/mp65Δ*::*HIS1 P_MET3_-MP65-GFP-URA3*
GZY934	BWP17, *CYR1/cyr1Δ::FRT*
GZY942	BWP17, *cyr1Δ*::*UFP/cyr1Δ*::*HIS1*
GZY1094	BWP17, *bcr1Δ*::*UFP/bcr1Δ*::*HIS1*
GZY1512	BWP17, *sln1Δ*::*UFP/sln1*::*HIS1*
GZY1513	BWP17, *sln1Δ*::*FRT/sln1*::*HIS1*
GZY1518	BWP17*, sln1Δ*::*FRT/sln1*::*HIS1 SLN1-Myc-URA3*
GZY1520	BWP17, *sln1Δ*::*FRT/sln1*::*HIS1 TetOff-Myc-EFG1-URA3*
GZY1521	BWP17, *mp65Δ*::*FRT/mp65Δ*::*HIS1 TetOff-Myc-EFG1-URA3*
GZY1519	BWP17, *cyr1Δ::FRT/cyr1::P_MET3_-Myc-CYR1-URA3*

**TABLE 2:** Plasmid constructs used in this study

Construct	Description
CIP10U	C. albicans integration vector with *URA3* as selection marker; generated by removing *RP10* gene from CIp10
CIP10H	C. albicans integration vector with *HIS1* as selection marker; *HIS1* was cloned into CIP10U at NotI and MluI to replace *URA3*
CIP10A	C. albicans integration vector with *ARG4* as selection marker; *ARG4* was cloned into CIP10U at NotI and MluI to replace *URA3*
pYGS1002	LYS2ΔUFP-1/pBKS; *LYS2* promoter region (bp −500 to −1) and terminator region (bp 4216 to 4615) were amplified by PCR and cloned into vector pBKS at KpnI-XhoI and NotI-SacII, respectively, to flank UFP, located between XhoI and NotI; knockout cassette was released by KpnI and SacII for transformation to generate *lys2*Δ::*FRT*
pYGS1003	LYS2ΔUFP-2/pBKS; *LYS2* C-terminal coding region (bp 3761–4215) was amplified by PCR and cloned into pYGS1002 at NotI-SacII to replace terminator region (bp 4216 to 4615); the knockout cassette was released by KpnI and SacII for transformation to generate *lys2*Δ::*FRT*
pYGS1025	P_MET3_-Myc-UTR/CIP10U; *MET3* promoter (bp −1384 to −1) was PCR amplified and cloned (between KpnI and XhoI) in front of an N-terminal 6×*Myc* epitope (between XhoI and ClaI), followed by *UTR* (3′ untranslated region of *CaGAL4*, between ClaI and PstI), into CIP10U; the plasmid was linearized by SalI within *MET3* promoter for integration into GZY790 to generate GZY808
pYGS1029	P_MET3_-Myc-CYR1n/CIP10U; *CYR1* N-terminal region (bp 4–600) was PCR amplified and cloned into pYGS1025 at ClaI-PstI to replace *UTR*; the plasmid was linearized by PflMI within *CYR1n* for integration into GZY790, BWP17 and GZY934 to generate GZY809, GZY835 and GZY1519, respectively
pYGS1033	CYR1ΔUFP/pBKS; *CYR1* promoter region (~500 bp) and terminator region (~450 bp) were amplified by PCR and cloned into the vector pBKS at KpnI-XhoI and NotI-SacII, respectively, to flank *UFP*; the knockout cassette was released by KpnI and SacII for transformation to generate *cyr1*Δ::*UFP* and *cyr1Δ::FRT*
pYGS1066	MP65c-HA-UTR/CIP10H; the C-terminal coding region of *MP65* (bp 50–1134) was PCR amplified and cloned (between KpnI and XhoI) in front of a C-terminal 2×*HA* epitope (between XhoI and ClaI), followed by *UTR* (between ClaI and PstI), into CIP10H; the plasmid was linearized by HpaI within *MP65c* for integration into BWP17 and GZY835 to generate GZY865 and GZY866, respectively
pYGS1068	MP65ΔUFP/pBKS; *MP65* promoter region (bp −500 to −1) and terminator region (bp 1138 to 1589) were amplified by PCR and cloned into vector pBKS at KpnI-XhoI and NotI-SacII, respectively, to flank *UFP*; the knockout cassette was released by KpnI and SacII for transformation into BWP17 to generate *mp65Δ*::*UFP* and *mp65Δ*::*FRT*
pYGS1070	MP65ΔHIS1/pBKS; *HIS1* was amplified by PCR and cloned into pYGS1068 at XhoI-NotI to replace *UFP*; the knockout cassette was released by KpnI and SacII for transformation to generate *mp65Δ*::*HIS1*
pYGS1085	P_MET3_-MP65-GFP-UTR/CIP10U; *MP65* (bp 1–1134) was amplified by PCR and cloned at BamHI-XhoI to under *MET3* promoter (KpnI-BamHI) control, and in front of a C-terminal *GFP* epitope (XhoI-ClaI), followed by *UTR* (ClaI-PstI), into CIP10U; the plasmid was linearized by NsiI within *MET3* promoter for integration into GZY888 to generate GZY900
pYGS1121	CYR1ΔHIS1/pBKS; *HIS1* was amplified by PCR and cloned into pYGS1033 at XhoI-NotI to replace *UFP*; the knockout cassette was released by KpnI and SacII for transformation to generate *cyr1*Δ::*HIS1*
pYGS1249	TetOff-Myc-EFG1n-UTR-TetR/CIP10U; the tetracycline operator sequence (*TetOff*) was cloned into CIP10U at KpnI-XhoI to control expression of a 6×*Myc* epitope (cloned at XhoI-ClaI); the N-terminal coding region of *EFG1* (bp 4–1500) was PCR amplified and cloned at ClaI-PacI to fuse in frame with the 6×*Myc* epitope and followed by *UTR* (at PacI-PstI); the tetracycline-repressible transactivator (*TetR*) was cloned after *UTR* at PstI-MluI; the plasmid was linearized by XcmI within *EFG1n* for integration to overexpress *EFG1* in the absence of doxycycline
pYGS1250	BCR1ΔUFP/pBKS; *BCR1* promoter region (~500 bp) and terminator region (~450 bp) were amplified by PCR and cloned into vector pBKS at KpnI-XhoI and NotI-SacII, respectively, to flank *UFP*; the knockout cassette was released by KpnI and SacII for transformation into BWP17 to generate *bcr1*Δ::*UFP*
pYGS1251	BCR1ΔHIS1/pBKS; *HIS1* was amplified by PCR and cloned into pYGS1250 at XhoI-NotI to replace *UFP*; the knockout cassette was released by KpnI and SacII for transformation to generate *bcr1*Δ::*HIS1*.
pYGS1495	SLN1ΔUFP/pBKS; *SLN1* promoter region (~500 bp) and terminator region (~450 bp) were amplified by PCR and cloned into vector pBKS at KpnI-XhoI and NotI-SacII, respectively, to flank *UFP*; the knockout cassette was released by KpnI and SacII for transformation into BWP17 to generate *sln1*Δ::*UFP* and *sln1*Δ::*FRT*
pYGS1496	SLN1ΔHIS1/pBKS; *HIS1* was amplified by PCR and cloned into pYGS1495 at XhoI-NotI to replace *UFP*; the knockout cassette was released by KpnI and SacII for transformation to generate *sln1Δ*::*HIS1*
pYGS1499	SLN1-Myc-UTR/CIP10U; promotor and coding region of *SLN1* gene was PCR amplified and cloned into CIP10U between AscI and XhoI in frame with a C-terminal *Myc* epitope (followed by *UTR*). The plasmid was linearized by BglII within the promoter region for integration

For filamentous growth on Spider plates, yeast cells were inoculated into YPD and cultured at 30°C overnight. Cells were then streaked onto plates to form single colonies by incubation at 30°C for 6 days. Images of filaments along a colony edge were taken using a Leica LEITZ DM RB optical microscope equipped with a Moticam 10MP digital camera. For filamentous growth on serum and RPMI plates, YPD cultures were diluted with water to an optical density at 600 nm (OD_600_) of 1.0, and 5 μL of the diluted culture was spotted onto the plate followed by incubation at 37°C for 4 to 5 days. Images of filamentation were taken using a Leica MZ 16 F optical microscope equipped with the Moticam 10MP digital camera. For hyphal induction, the YPD culture was 1:20 diluted into fresh YPD containing 10% fetal bovine serum (HyClone) and incubated at 37°C for 2 h. Cells were visualized with a Leica DMRXA2 microscope equipped with a Coolsnap HQ2 digital camera. Images were acquired using MetaMorph 7.5 software.

For the Congo red sensitivity test, yeast cells were grown to the stationary phase in YPD at 30°C for 2 days. The cell suspensions were then serially diluted (1:10), spotted onto YPD plates containing 100 μg/mL of Congo red (Sigma-Aldrich), and incubated at 30°C for 24 h.

### Protein extraction, IP, and WB.

To prepare protein extracts, cells were harvested into 2-mL screw-cap microcentrifuge tubes by brief centrifugation to obtain pellets with a volume of ≤500 μL and resuspended in an equal volume of ice-cold lysis buffer (50 mM Tris-HCl [pH 7.4], 150 mM KCl, 1% NP-40) containing protease inhibitor cocktail (Nacalai Tesque) and phosphatase inhibitor cocktail B (Santa Cruz). After adding an equal volume of acid-washed glass beads (Sigma-Aldrich), cells were broken by 5 rounds of 60-s beating at 5,000 rpm in a TOMY Micro Smash beater (MS-100) with 1 min of cooling on ice between rounds. The lysed cells were then centrifuged at 14,000 rpm for 15 min at 4°C to collect the supernatant.

To perform IP, cell lysates were incubated with 30 μL of a slurry of goat polyclonal HA (Santa Cruz) or Myc (Santa Cruz) beads at 4°C for ≥1 h. After brief centrifugation at 8,000 rpm, the beads were washed by resuspension in 800 μL cold lysis buffer, followed by another brief centrifugation. The wash was repeated 4 more times, and beads were finally resuspended in 10 μL 1× protein loading buffer (50 mM Tris-HCl [pH 6.8], 2% SDS, 10% glycerol, 1% β-mercaptoethanol, and 0.02% bromophenol blue) and boiled for 8 min. Protein samples were separated by SDS-PAGE and transferred to a polyvinylidene difluoride membrane (Bio-Rad).

For WB analysis, the membrane was first incubated with 5% milk in phosphate-buffered saline solution containing 0.1% Tween 20 (PBST) at room temperature for 1 h or at 4°C overnight. After a brief rinse with PBST, the membrane was incubated in PBST containing a 1:1,000 diluted mouse monoclonal HA (Santa Cruz) or Myc (Santa Cruz) antibody at room temperature for 1 h, followed by 3 rounds of a 5-min wash with PBST. The membrane was then incubated in PBST containing a 1:2,500-diluted secondary antibody (horseradish peroxidase-linked anti-mouse IgG from sheep; GE Healthcare). After 3 rounds of a 5-min wash with PBST, the membrane was immersed in ECL Western blotting substrate solution (Thermo Scientific) and exposed to X-ray film (Fujifilm).

### Sample preparation for SILAC incorporation assay.

GZY790 cells were inoculated into 2 mL of GMM supplemented with histidine, uridine, arginine-d10 (Cambridge Isotopes), and lysine-d8 (Cambridge Isotopes) (heavy medium) and grown at 30°C overnight. The culture was reinoculated into 20 mL of the heavy medium and grown at 30°C for one more day. Cells were then harvested, and protein extract was prepared with urea lysis buffer (20 mM HEPES [pH 8.0], 9 M urea, 1 mM sodium orthovanadate, 2.5 mM sodium pyrophosphate, and 1 mM β-glycerophosphate). MS samples were prepared using the filter-aided sample preparation (FASP) protocol (65) with some modifications. Briefly, a 1:10 (vol/vol) solution of 50 mM dithiothreitol (DTT) was added to the lysate (100 μg) and incubated at 60°C for 20 min with shaking. Urea at 8 M in 0.1 M Tris-HCl (pH 8.5; UA) was added to the lysate and transferred to a Millipore 30-kDa Amicon filter unit. Buffer exchange was done on this filter unit with UA before alkylation with 50 mM iodoacetamide (IAA) in the dark for 20 min. Trypsin (porcine, modified sequencing grade; Promega) digestion (1:100) in 40 mM ammonium bicarbonate (ABC; Sigma-Aldrich) was performed on the filter at 37°C overnight. The next day, peptides were eluted by centrifuging at 20,000 × *g* for 10 min at room temperature, with additional elution using 40 mM ABC. Total tryptic-digested peptides were concentrated using a speed vacuum concentrator before introducing them into the mass spectrometer for incorporation analysis.

### Immunopurification of Myc-Cyr1-associated proteins.

The strain expressing Myc-Cyr1 (GZY809) was inoculated into 50 mL of the heavy medium and grown at 30°C overnight. The culture was reinoculated into 500 mL of the heavy medium and grown at 30°C for one more day. The control strain GZY808 was cultured similarly in the light medium (GMM supplemented with histidine, uridine, arginine, and lysine). Cells were then harvested into 2-mL screw-cap microcentrifuge tubes, and 7.5 mL of heavy and light protein extracts were prepared and incubated with 40 μL EZview red anti-Myc affinity gel (Sigma-Aldrich) at 4°C for 4 h. After washing with 1 mL of lysis buffer twice, the beads were combined and washed 3 more times, finally resuspended in 40 μL of 1× protein loading buffer, and boiled for 8 min. Eluted protein complexes were separated by one-dimensional 4 to 12% NuPage Novex bis-Tris gel (Invitrogen) and stained using the colloidal blue staining kit (Invitrogen). All visualized protein bands were excised for quantitative proteomic analysis.

### Quantitative MS.

SDS-PAGE-separated proteins were digested in-gel with trypsin using published protocols (66). Peptide samples were analyzed on an Orbitrap (Thermo Fisher). Survey full-scan MS spectra (*m/z* 310 to 1400) were acquired with a resolution of *r* = 60,000 at *m/z* 400, an automatic gain control target of 1e6, and a maximum injection time of 500 ms. The 10 most intense peptide ions in each survey scan with an ion intensity of >2,000 counts and a charge state of ≥2 were isolated sequentially to a target value of 1e4 and fragmented in the linear ion trap by collision-induced dissociation using a normalized collision energy of 35%. Dynamic exclusion was applied using a maximum exclusion list of 500 with one repeat count, repeat, and exclusion duration of 30 s. Raw data were searched using C_albicans_SC5314_version_A21-s02-m01-r02_orf_trans_all.fasta. Database searches were performed with tryptic specificity allowing a maximum of two missed cleavages and two labeled amino acids and an initial mass tolerance of 6 ppm for precursor ions and 0.5 Da for fragment ions. Cysteine carbamidomethylation was searched as a fixed modification, and N-acetylation and oxidized methionine were searched as variable modifications. Labeled arginine and lysine were specified as fixed or variable modifications, depending on the prior knowledge about the parent ion. SILAC peptide and protein quantification was performed with MaxQuant version 1.2.0.18 using default settings. Maximum false-discovery rates were set to 0.01 for both protein and peptide. Proteins were considered identified when supported by at least one unique peptide with a minimum length of 6 amino acids.

### Biofilm development and quantification.

The biofilm development assay was performed using a method described previously with slight modifications (67). Single colonies were inoculated in liquid GMM and incubated at 30°C overnight with shaking at 180 rpm. Cells were washed twice with PBS. The cell concentration was adjusted to 10^7^ cells/mL of PBS by using a haemocytometer. A 100-μL aliquot of the cell suspension was transferred to a well of a flat-bottomed 96-well polystyrene plate (tissue culture treated; Falcon) and incubated for 1.5 h at 37°C with shaking at 75 rpm to allow cell adherence. The plate was sealed with the a Breathe-Easy sealing membrane (Axygen) to prevent cross-contamination between wells and edge effect. Next, the nonadherent cells were removed, and the well was washed twice with 150 μL of PBS. A 200-μL volume of fresh GMM was added, and the mixture was incubated for 48 h at 37°C with shaking at 75 rpm. Medium was changed after 24 h to remove planktonic cells. After 48 h of incubation, planktonic cells were aspirated from the wells and the biofilms were washed twice with PBS prior to quantification via the OD_600_ and metabolic activity measurement.

For biofilm visualization, the 96-well polystyrene plate was mounted on the stage of an inverted microscope after drying of the biofilm. High-magnification images of C. albicans cells were taken with an Olympus IX70 microscope equipped with a Motic Cam 2.0 MP camera and Motic Images Plus 2.0 software.

For biofilm measurement, the OD_600_ was read using a microplate reader (Tecan Infinite M200 Pro). Five independent locations per well were read to gain an averaged density. The metabolic activity of the biofilms was measured using the 2,3-bis-(2-methoxy-4-nitro-5-sulfophenyl)-2H-tetrazolium-5-carboxanilide, disodium salt (XTT) colorimetric assay (Biotium) according to the manufacturer’s protocol. A 150-μL volume of PBS and 25 μL of activated XTT solution (activation reagent:XTT solution ratio, 1:200) was added in each well. The plate was then incubated at 37°C for 2 h with shaking in the dark. A 100-μL aliquot of the supernatant was transferred to a new well. The absorbance was read at 490 nm using a microplate reader. Graph Pad Prism software version 6.00 was used for all statistical analyses. Data are expressed as means ± standard deviations. Results of the biofilm development assay were analyzed by two-tailed unpaired *t* test. All experiments were repeated at least three times independently.
