# Supplementary material for: Comprehensive Interactome Analysis for the Sole Adenylyl Cyclase Cyr1 of Candida albicans
Source: Microbiol Spectr. 2022 Oct 31;10(6):e03934-22. doi: 10.1128/spectrum.03934-22 (PMC9769623; doi:10.1128/spectrum.03934-22)
Supplement: Supplemental file 2 — Fig. S1. Download spectrum.03934-22-s0002.pdf, PDF file, 0.2 MB [file spectrum.03934-22-s0002.pdf]

Figure S1

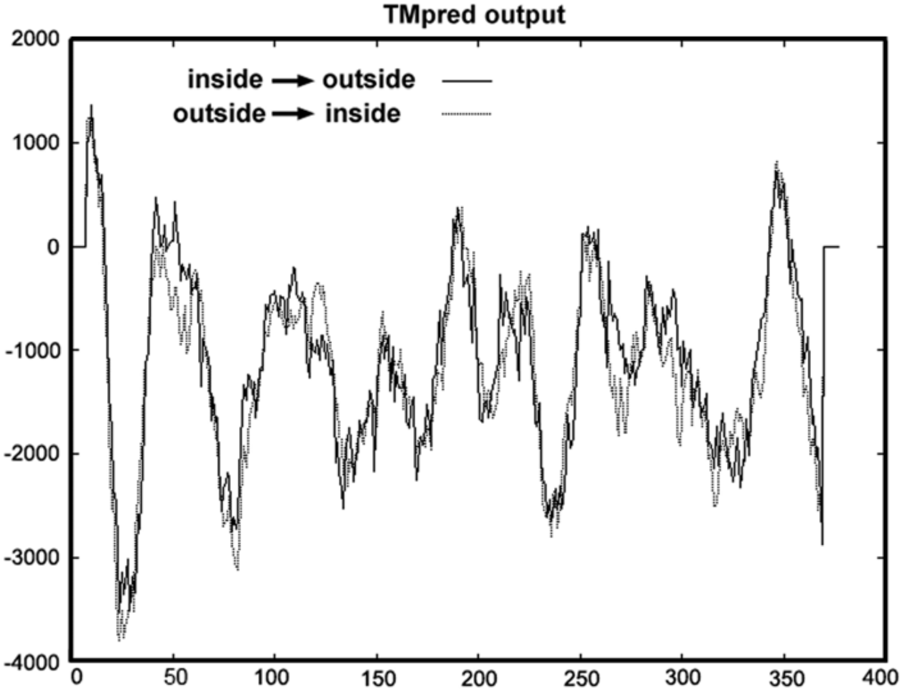

2 possible models considered, only significant TM-segments used

STRONGLY prefered model: N-terminus inside
2 strong transmembrane helices, total score : 2175

#	from	to	length	score	orientation
1	1	20	(20)	1367	i-o
2	338	361	(24)	808	o-i

Alternative model
2 strong transmembrane helices, total score : 1962

#	from	to	length	score	orientation
1	1	21	(21)	1247	o-i
2	338	357	(20)	715	i-o

Figure S1: Highly possible transmembrane domains in Mp65 identified by the transmembrane topology analysis. Regions exposed to both the outside and inside of the plasma membrane are listed.
